# Aiming for long-term, objective-driven science communication in the UK

**DOI:** 10.12688/f1000research.9079.2

**Published:** 2016-12-08

**Authors:** Andreas Prokop, Sam Illingworth

**Affiliations:** 1 Faculty of Life Sciences, The University of Manchester, Manchester, M13 9PT, UK; 2 School of Research, Enterprise & Innovation, Manchester Metropolitan University, Manchester, M1 5GD, UK

**Keywords:** Science communication, funding, natural sciences

## Abstract

Communicating science to wider lay audiences is an increasingly important part of a scientist's remit, and is something that many scientists are keen to embrace. However, based on surveys carried out amongst the UK public, as well as our own experiences in developing and delivering such activities, we believe that they are not always as effective at engaging members of the general public as they could be. In this opinion article we argue that in order to achieve more effective science communication, we need more objective-driven and long-term initiatives. As well as being implemented by the scientists themselves, funding organisations can play an important role in helping to drive such initiatives, and we suggest a list of actionable items that might allow for some of these ideas to be implemented.

## Potential shortcomings in UK engagement

The purpose of science communication is to “enhance public scientific awareness, understanding, literacy, and culture” ( Burns *et al.*, 2003, pp. 198), and many funding bodies in the UK (and indeed worldwide) made it an obligation, over a decade ago, that researchers actively engage with lay audiences and communicate their research to them (see e.g. Holbrook, 2005). However, even a decade later, the ‘Public Attitudes to Science’ survey in the UK, conducted by Ipsos MORI on behalf of the British Science Association ( Castell *et al.*, 2014), reported a clear shortfall amongst the general public in terms of science communication, with only 45% of respondents (n=1749, age >16) feeling aware of science in general and 51% stating they received too little information. Similarly, the ‘Factors affecting public engagement by researchers’ study by the Wellcome Trust ( Hamlyn *et al.*, 2015) found that public engagement is more firmly embedded in the culture of the arts, humanities and social sciences than it is among researchers in Science, Technology, Engineering and Mathematics (STEM) - in spite of the fact that the majority of scientists are still keen to engage, with only 1% of STEM researchers (n=1556) stating that they would like to spend *less* time engaging with the public. The results of these surveys would seem to suggest that STEM researchers are not entirely successful at engaging the public with their research.

Here we argue for a need to re-think the ways in which we communicate science; that we require a long-term and objective-driven vision, and that support is required to ensure that initiatives incorporating such a vision, and the people who drive them, are given the opportunity to succeed.

## Why engage long-term and with clear objectives?

How long-term strategies and clear objective-setting can lead to impact is nicely illustrated by a number of exceptional initiatives of science communication. For example, *Zoouniverse* is a long-term initiative encompassing numerous citizen science projects, and thus providing opportunities for non-experts around the world to help to contribute towards real scientific discoveries (see e.g. Cox *et al.*, 2015). *EuroStemCell* ( http://www.eurostemcell.org) began as a science communication initiative for a European research consortium, and has since developed into an independent hub communicating the importance and value of stem cell research, and its web resources have become a central point of reference for the public and researchers alike. *Understanding Animal Research* ( http://www.understandinganimalresearch.org.uk) is a multi-facetted science communication initiative promoting and improving the acceptance of animal use in research, engaging with a variety of audiences using bespoke communication methods. The *iBiology* project generates films that explain in simple terms how science is conducted, thereby promoting a wider excitement about modern biology and an understanding of the processes by which scientific discoveries are made ( Goodwin, 2014). Finally, the *Manchester Fly Facility* promotes the use of fruit flies in the biomedical sciences through science fairs, YouTube videos, student training strategies, and a dedicated school programme developing biology lessons - with all of its resources made publicly and freely available ( Patel & Prokop, 2015).

All of these examples represent long-term initiatives driven by an overarching vision or objective. As is evident from the success of these projects, such an approach offers a number of important advantages:

First, long-term strategies provide enough time to develop a broader range of activities (e.g. science fairs, art-science collaborations, engagement in schools, citizen science, development of online resources etc.) and, with it, a widening of potential audiences and impact (see e.g. Patel & Prokop, 2015; Silvertown, 2009).

Second, long-term initiatives allow for gradual and cumulative developments, including the generation of high-quality activities and resources. Once strategies and resources have been generated and shared online, they can develop their own dynamics (e.g. web resources being viewed by the public; shared strategies being applied by fellow scientists anywhere in the world) and save preparation time for future events, thereby making initiatives more impactful and sustainable.

Third, long-term strategies make it possible to incorporate interdisciplinary expertise. For example, artists can help to improve the appeal and interactivity of an activity, and social scientists or specialists in science communication can facilitate strategy improvement in a number of ways: by helping to advance beyond the "deficit model" of communication and engaging audiences via two-way dialogue ( Bucchi, 2008), by advising on formative research to guide objective setting ( Davies, 2008; Nisbet & Scheufele, 2009), and by assisting with frame-setting and media work ( Bubela *et al.*, 2009). Furthermore, such specialists can contribute expertise in production and project management, marketing, audience dynamics, and target audience-specific strategies.

Fourth, long-term initiatives provide room for the implementation of effective evaluation strategies ( Jensen, 2014), which should ideally go beyond simply the collection of basic metrics and demographics, and should also aim to monitor gradual developments over time. For example, if a science communication project has the clear objective of improving the knowledge of different climate change mitigation strategies in a local community, this specific knowledge can be measured at different stages of the project to evaluate success and progress.

Finally, long-term initiatives make it more likely that science communication will be of professional and personal benefit to the engaging scientists. It can enhance a professional profile within scientific communities and add to promotion portfolios. For young researchers, the additional skills in communication, teaching, project and people management can help to make them stand out from their contemporaries in terms of future grant and job applications ( Illingworth & Roop, 2015), or offer fantastic opportunities to transcend the academic environment by developing important transferable skills, such as didactic qualities for future teachers, or experiences with production, press, marketing and audience dynamics for those wanting to work in the media.

## Improving science communication: overcoming barriers

As can be seen from the above discussion, long-term and objective-driven science communication can have a wide-range of potential benefits for a variety of stakeholders (scientists, general public, interdisciplinary collaborators, etc.). However, successful implementation of such initiatives requires stamina, belief, dedication and time. This poses important individual barriers to scientists who are attempting to communicate their research, in particular the lack of time, but also issues of self-perception (i.e. status, competence) and attitude, such as the view that outreach is subsidiary to research and university teaching ( Hamlyn *et al.*, 2015). We feel that there are a number of further barriers which stand in the way of long-term and objective-driven science communication and which cannot be overcome by individuals or smaller collectives themselves. We believe that important encouragement and support could come directly from policy and decision makers who ultimately determine the portfolio and direction of research in the UK, but in particular also from the various UK funding organisations, which are in a powerful position to drive such developments. We will outline some of the most important barriers to long-term and objective-driven science communication, and make a number of actionable suggestions via which to overcome them:

**First,** it is important to develop a culture where objective-driven, long-term science communication has a fair chance of receiving the necessary support, to not threaten the enormous time and effort already invested into those initiatives that have gone a good distance and maintain a clear vision and pathway towards further impact. It is treading a thin line keeping the funding between spectacular one-off events, newly developed projects or initiatives (as the breeding ground for creative innovation), as well as of long-term science communication initiatives. Many funders consider support of ongoing activities in their guidelines already, but it requires awareness and vigilance to maintain a productive balance that will foster a vibrant culture of science communication.

**Second,** developing a culture of objective-driven, long-term science communication initiatives requires wide-spread sharing of and training in best practice. Funding organisations could further facilitate the instalment of public engagement officers helping scientists develop more powerful science communication strategies and providing urgently needed training. It could also become a more widely used practice that professional science communicators join science funding committees - not only so that content and strategies of proposed science communication projects benefit from a more balanced view, but also to give constructive feedback to applicants, advising them on more efficient communication strategies. Furthermore, an audit in the guise of the impact case studies from the UK’s last Research Excellence Framework exercise ( Illingworth & Roop, 2015) might be helpful to connect science communication activities and to assess which of the current science communication initiatives would most benefit from further funding.

**Third,** developing long-term initiatives requires an efficient dissemination of information, resources and strategies to specific target audiences and between scientists. However, the advent of the internet and social media has resulted in a flood of information that can make it surprisingly difficult to communicate and disseminate. Here, funders could help by using their dissemination means to raise awareness of good initiatives of science communication. Analogous to lobbying for open access (e.g. Harnad *et al.*, 2004), funders could also use their influence to change journal policies towards being more receptive to the publication of science communication articles - which is the perhaps most powerful way to reach fellow scientists, provides an important path towards professional reward and further justification for the public money invested into those initiatives.

**Finally,** one of the most substantial barriers to objective-driven, long-term science communication initiatives is a lack of external recognition and reward by local line managements ( Andrews *et al.*, 2005; Ecklund *et al.*, 2012), which tend to operate under financial and political pressures that are driven by performance in research and higher education. Initiatives that may have started with great enthusiasm will eventually have to face a reality-check based on the criteria of promotion or recruitment committees. Despite the opportunities that science communication can create, involvement in science communication is often viewed by scientists to be more damaging to their careers than helpful, as found in the Wellcome survey: “research suggests that researchers and institutions remain uncertain about systems of rewards for public engagement” ( Hamlyn *et al.*, 2015, pp. 5). In order to tackle this problem, funders could collaborate to drive the development of a national professional framework for science communication, which could guide funders, institutions and researchers towards implementation of best practice and effective local protocols - including also professional reward and recognition for public engagement as a crucial motivator. However, we note that such a framework should avoid stratification and aim to be inclusive of all levels in terms of science communication experience.

The idea of collaboration between funders is certainly not new. For example, the Concordat for Engaging the Public with Research contained a comprehensive list of desirable standards and practices ( RCUK, 2010) and was signed by an impressive list of UK science funders. Unfortunately, it did not include a concrete implementation strategy that could have driven it to impact. Similarly, the Beacons for Public Engagement Initiative (2008 to 2012) was funded by the four UK Funding Councils, Research Councils UK and the Wellcome Trust. It gave rise to the National Co-ordinating Centre for Public Engagement (NCCPE) providing new resource and capacity for public engagement ( Duncan & Manners, 2012); but the initiative did not lead to a long-term and effective harmonisation or alignment of public engagement strategies across the contributing organisations. These examples illustrate that serious collaboration between funding organisations could be achieved, and this would enormously facilitate many of the aforementioned actionable items and promote common strategies and objectives for science communication. In an ideal world, this could go as far as setting up a common fund for science communication, which would be easier to shield from organisation-specific objectives and policies, thus facilitating the development of overarching funding models that set new standards for science communication nationwide.

## Final thoughts

This article is an opinion piece, based on the experience of two long-standing but very different personal science communication histories and backgrounds. We recognised that there is an astonishing congruence in views and experiences, and were also strongly encouraged by the very positive comments from competent colleagues (see acknowledgements), and by the numerous in-depth discussions we had with them. We feel passionately about the need to improve standards and to instil a solid culture of science communication, and have discussed the enormous opportunities provided by long-term and objective-driven initiatives to this end. We have expressed our opinion that bottom-up implementation driven by scientists will only go so far. We need more support from funding bodies tailored towards overcoming barriers that stand in the way of desirable developments towards a solid culture of science communication where efforts can be aligned at all levels including scientists, local institutions, funding organisations and policy makers in the UK - and similar may be true also in other countries.

Independent bodies such as the British Science Association or the NCCPE could help as facilitators during this process, capitalising on experiences for example from the Beacons initiative. Perhaps even government involvement is required to guide the process, building on the code of conduct by the Council for Science and Technology, which stated the need for scientists to communicate their research to the wider society ( Poliakoff & Webb, 2007). Their implementation might be most effective at the institutional level, for example using compliance as a factor that impacts on funding allocations, as has been successfully used by the Athena SWAN charter to address gender equality ( Donald *et al.*, 2011).

We hope that our thoughts provoke useful discussion about future ways forward and encourage those who already follow good practice to make themselves heard.
