# Supplementary material for: Aiming for long-term, objective-driven science communication in the UK
Source: F1000Res. 2016 Dec 8;5:1540. Originally published 2016 Jun 29. [Version 2] doi: 10.12688/f1000research.9079.2 (PMC5270583; doi:10.12688/f1000research.9079.2)
Supplement: Peer review history

# Peer review: Referee response for version 2

**DOI:** 10.5256/f1000research.11083.r18348

**Published:** 2017-01-26

**Authors:** Massimo Caine

**Affiliations:** 1 Microbiology Unit, Department of Botany and Plant Biology, University of Geneva, Geneva, Switzerland

I have read the revised article.

 The authors have expanded the manuscript from the comments previously raised and have proposed a new version indeed more focused on the issues that they wish to address.

 However, I must say that, overall, I hardly find the spark of novelty that should lead the reader to take action based on what is presented. Personally, as interested reader and (ideally) a potential funder, I would have liked to better understand what have made successful the UK-example described ( *e.g. *impact evaluation, audience deliverables) so to better frame the examples within the long-term/objective driven funding discussion (which is, in the end, the main objective of the current submission). Also, on a different but related note, reducing the description of the barriers to (i) stakeholders commitment and (ii) funds availability lacks to address some other quite important barriers that would have worth to be mentioned ( *e.g.* government/political agenda, academic curricula, peers support etc.). This could have been expanded as well for the sake of discussion.

 Given my hesitation concerning the above-mentioned issues, I am accepting the manuscript with reservation (and with apologies for the delay). This, to my understanding of the F1000 reviewing process, will make the article ready to be indexed.

I have read this submission. I believe that I have an appropriate level of expertise to confirm that it is of an acceptable scientific standard, however I have significant reservations, as outlined above.

# Peer review: Referee response for version 2

**DOI:** 10.5256/f1000research.11083.r18349

**Published:** 2017-01-03

**Authors:** Viviane Callier

**Affiliations:** 1 Developmental Physiology, Ronin Institute, Montclair, NJ, USA

I have now read the revised article.

 The revision is a significant improvement on the first version, in that the authors provide specific examples of science communication initiatives and have better organized the text.

 The main message, if I understand it correctly, is that policy makers and science funders need to provide resources and the right incentives to support a strong culture of science communication. While true, I don't know that this idea is particularly novel -- and with funding agencies' budgets so constrained now, it's not clear to me whether they'll be sold on creating a special science communication fund.

 The authors list several challenges of science communication related to resources and incentives, but don't really talk about the challenges inherent to science communication that would remain if such resources/incentives were available. I think this area might be a fruitful one to explore. For example:

1. Who is our audience, and how do we know when we've reached them? (Can you talk about challenges in measuring this, and propose ideas?)
2. What do we want them to *do* with the information being communicated? (Your article talks about "objective-driven" communication, but nowhere is it specified what the objectives or outcomes are!)
3. How do we measure whether the audience has received the message and done what we intended (e.g., was the objective accomplished)?

These are the central questions to me, and the really critical ones for any science communicator to clarify before embarking on a science communication project.

 Although I did not find the piece to make novel points, I did find the article much improved on the first version, and because tomorrow is Christmas I am marking it approved.

I have read this submission. I believe that I have an appropriate level of expertise to confirm that it is of an acceptable scientific standard.

# Peer review: Referee response for version 2

**DOI:** 10.5256/f1000research.11083.r18646

**Published:** 2016-12-20

**Authors:** Kathryn B. H. Clancy

**Affiliations:** 1 Department of Anthropology, University of Illinois, Urbana, IL, USA

I have now read the revised article.

 I’m still struggling to understand the importance of this opinion piece, and why it belongs in F1000. I am also still unclear on the purpose of telling this audience that long-term planning for science communication in the UK is important. I do happen to think these things are important, I’m not sure the authors are conveying these points in a way that will reach those who don’t feel this way.

 A few examples:

 The authors name a number of “exceptional initiatives of science communication,” then state “All of these examples represent long-term initiatives driven by an overarching vision or objective. As is evident from the success of these projects, such an approach offers a number of important advantages.” But they list five initiatives, they don’t spend any time describing why these initiatives are exceptional or effective. Then, the “important advantages” they list next are startlingly obvious: e.g. that long-term planning allows for time to develop “a broader range of activities,” and “graduate and cumulative developments” of programming.

 The authors identify some of the biggest barriers to science communication: a lack of a culture that supports it, and few incentives for scientists. But again, this isn’t novel so much as exactly the sort of thing science communicators discuss all the time. I also didn’t read the responses to these barriers as particularly novel, but again exactly the sorts of things we all bemoan all the time.

I have read this submission. I believe that I have an appropriate level of expertise to confirm that it is of an acceptable scientific standard, however I have significant reservations, as outlined above.

# Peer review: Referee response for version 1

**DOI:** 10.5256/f1000research.9772.r15848

**Published:** 2016-08-24

**Authors:** Kathryn B. H. Clancy

**Affiliations:** 1 Department of Anthropology, University of Illinois, Urbana, IL, USA

The opinions shared in this piece were undercited, vague, and not novel.

                                                   

 Title and abstract: The authors should consider a title that describes the take-home message of the article. They should also decide on one way of capitalizing the title (all or nothing).

 Article content: The organization of this paper was not clear, and it also wasn’t clear what this opinion piece was intended to do. Was this written towards funding organizations, individual scientists? What are the actionable items that might allow for a funding organization to test out some of these ideas? A conceptual model or figure would have greatly helped clarify this.

 Conclusions: The authors might get more traction by developing a more concrete conceptual model with testable hypotheses. This would also help them fund the work to show if their ideas would lead to improved science literacy among the general public.

I have read this submission. I believe that I have an appropriate level of expertise to confirm that it is of an acceptable scientific standard, however I have significant reservations, as outlined above.

# Response

**Published:** 2016-11-18

**Authors:** Andreas Prokop

**Affiliations:** The University of Manchester, UK

What have we changed in this version?

 This version of the opinion piece has been substantially edited to make the message clearer that we are trying to communicate, i.e. the need for objective-driven, long-term science communication in the UK. We have streamlined these arguments, and present now more coherently the potential barriers to such a vison, as well as actionable suggestions for how to overcome them. We have also changed the structure and title of the paper to be more fitting to the content, and make it much clearer from the outset that this is an opinion piece and not a research article. Please, see our detailed responses below. We look forward to our further discussion.

 Response to Reviewer 1

*Title and abstract: The authors should consider a title that describes the take-home message of the article. They should also decide on one way of capitalizing the title (all or nothing).*

  

- Thank you for your comments. We take your points regarding the title, and as such have modified it accordingly.
* *

*Article content: The organization of this paper was not clear, and it also wasn’t clear what this opinion piece was intended to do. Was this written towards funding organizations, individual scientists? What are the actionable items that might allow for a funding organization to test out some of these ideas? A conceptual model or figure would have greatly helped clarify this.*

*Conclusions: The authors might get more traction by developing a more concrete conceptual model with testable hypotheses. This would also help them fund the work to show if their ideas would lead to improved science literacy among the general public.*

*The opinions shared in this piece were under cited, vague, and not novel.*

  

- Organisation and objective: The paper has been edited so that it is now much clearer in terms of its objectives, and at your suggestion we have included a list of actionable items that we believe would be of benefit to funding organisations. In relation to a conceptual model with a testable hypothesis this is, in our view, not something that would sit well in an opinion piece. However, we now present some actionable items as potential solutions to overcoming the barriers that we present. In regards to the audience of this article, please, see the next bullet point.

 

- Novelty and target audience: This article is less aimed at academic science communicators than at STEM scientists who engage in science communication and at funding bodies that support STEM research. The aim is not to present novel ideas (although these thoughts will be novel to most STEM scientists working at the coal-face of science communication), but to alert to shortcomings and reinforce thinking about possible actionable ways in which to improve on this, many of which are being underused now or have been thought about but were never properly implemented (see last paragraph of section 3).
- Citations and vagueness: We believe that this version of the manuscript has addressed these issues. We are much clearer in the scope of the paper (identification of problem, context for why it exists, and potential solution(s)), and are also more rigorous in our use of referencing. We have now included a paragraph providing concrete examples of long-term objective-driven initiatives. We also believe that some of the solutions that we propose are novel, and that this is now easier to see given the edit of the paper.

# Peer review: Referee response for version 1

**DOI:** 10.5256/f1000research.9772.r14993

**Published:** 2016-08-09

**Authors:** Massimo Caine

**Affiliations:** 1 Microbiology Unit, Department of Botany and Plant Biology, University of Geneva, Geneva, Switzerland

This opinion article seeks to describe standards, barriers and possible initiatives that may be relevant for the development of science communication in the UK. Even if opinions concerning a certain topic (in this case science communication) may be very wide and call into consideration several aspects of the subject, I find that the manuscript lacks a clear focus, which, in turn, makes the flow of the text very hard to follow.

 In particular, in the initial part of the manuscript, authors state that they “will discuss the current practice of science communication in the UK”. However, despite a very general description of the relevance of science communication practice for (i) policy making; (ii) education; (iii) scientific networking and (iv) professional development, there is a substantial lack of any description of experience, best practice, event or initiative that may have taken place in the UK. Again, when describing the several barriers that hamper a proper diffusion and development of science communication, the observations are rather general, superficial and seemingly not focused on the UK scenario (at least this is the perception I have as non-UK reader). Consequently, the suggestions on how to overcome such barriers are extremely hard to be contextualized within and they seem to me rather focused on funding agencies and their policies rather than on the good practice/development of science communication.

 In light of that, despite the good will that authors put in place for the cause of science communication, I regret to say that I cannot recommend this opinion article to be indexed as it is.

I have read this submission. I believe that I have an appropriate level of expertise to state that I do not consider it to be of an acceptable scientific standard, for reasons outlined above.

# Response

**Published:** 2016-11-18

**Authors:** Andreas Prokop

**Affiliations:** The University of Manchester, UK

What have we changed in this version?

 This version of the opinion piece has been substantially edited to make the message clearer that we are trying to communicate, i.e. the need for objective-driven, long-term science communication in the UK. We have streamlined these arguments, and present now more coherently the potential barriers to such a vison, as well as actionable suggestions for how to overcome them. We have also changed the structure and title of the paper to be more fitting to the content, and make it much clearer from the outset that this is an opinion piece and not a research article. Please, see our detailed responses below. We look forward to our further discussion.

 Response to Reviewer 2

*This opinion article seeks to describe standards, barriers and possible initiatives that may be relevant for the development of science communication in the UK. Even if opinions concerning a certain topic (in this case science communication) may be very wide and call into consideration several aspects of the subject, I find that the manuscript lacks a clear focus, which, in turn, makes the flow of the text very hard to follow.*

  

- Thank you for your comments. As explained in our response to Reviewer 1, we concede that the central thesis may not have come across clearly enough. We have now re-focussed and edited the paper, and we hope that we make the main objective much clearer, i.e. that there is a problem (a lack of long-term, objective-led science communication), why this problem exists, and some suggested solutions for how we might best tackle this problem.

*In particular, in the initial part of the manuscript, authors state that they “will discuss the current practice of science communication in the UK”. However, despite a very general description of the relevance of science communication practice for (i) policy making; (ii) education; (iii) scientific networking and (iv) professional development, there is a substantial lack of any description of experience, best practice, event or initiative that may have taken place in the UK.*

  

- We agree that the statement about general practice did not match well with the material provided, and it has been removed. To provide concrete descriptions of good practice, we have added a whole paragraph on objective-driven long-term initiatives that are existing in the UK and beyond.

 

*Again, when describing the several barriers that hamper a proper diffusion and development of science communication, the observations are rather general, superficial and seemingly not focused on the UK scenario (at least this is the perception I have as non-UK reader). Consequently, the suggestions on how to overcome such barriers are extremely hard to be contextualized within and they seem to me rather focused on funding agencies and their policies rather than on the good practice/development of science communication.*


*  *

- To address the critique that comments and opinions that we expressed are too general and potentially over-reaching, we have tightened these up significantly and provide now a more rigorous justification for their inclusion. However, we have still chosen to focus on the role that funding agencies and their policies play, because we believe that they are in the best position to lower barriers and facilitate effective science communication.

# Peer review: Referee response for version 1

**DOI:** 10.5256/f1000research.9772.r14992

**Published:** 2016-08-04

**Authors:** Viviane Callier

**Affiliations:** 1 Developmental Physiology, Ronin Institute, Montclair, NJ, USA

I found the article to be written in vague, abstract terms, making it difficult to extract any take-home message. The problem that was to be solved wasn't clearly articulated, nor was the proposed "solution" clear to me. I found the article to be a poor example of science communication.

I have read this submission. I believe that I have an appropriate level of expertise to state that I do not consider it to be of an acceptable scientific standard, for reasons outlined above.

# Response

**Published:** 2016-11-18

**Authors:** Andreas Prokop

**Affiliations:** The University of Manchester, UK

What have we changed in this version?

 This version of the opinion piece has been substantially edited to make the message clearer that we are trying to communicate, i.e. the need for objective-driven, long-term science communication in the UK. We have streamlined these arguments, and present now more coherently the potential barriers to such a vison, as well as actionable suggestions for how to overcome them. We have also changed the structure and title of the paper to be more fitting to the content, and make it much clearer from the outset that this is an opinion piece and not a research article. Please, see our detailed responses below. We look forward to our further discussion.

 Response to Reviewer 3

*I found the article to be written in vague, abstract terms, making it difficult to extract any take-home message. The problem that was to be solved wasn't clearly articulated, nor was the proposed "solution" clear to me. I found the article to be a poor example of science communication.*

  

- The brevity of this comment makes it difficult to respond to and engage with in a detailed manner. However, we take the point that there were aspects of the opinion piece that were vague, and have addressed this thoroughly, as was explained above. We hope that the new version of the article now clearly describes the problem and the potential solution(s).

 

- If this is not the case, then we would like to ask the reviewer to please be a little bit more constructive in their criticism, so that we can improve the manuscript further.
